# Blood Pressure Control Among U.S. Adults With Hypertension, Stratified by Glucagon-Like Peptide 1 (GLP-1) Receptor Agonist Use: Evidence From the National Health and Nutrition Examination Survey, 2017-2020

**DOI:** 10.7759/cureus.113504

**Published:** 2026-07-28

**Authors:** Nadra Elfezzani, Theresa M Mambu, Jemie U Nnanna, Ikenna I Iwu, Blen W Nedassa, Eunice Nathan-Otu, Onize Ekome, Enemuo Idinmachukwu, Nagla A Massud, Azeberoje Osueni

**Affiliations:** 1 Medicine, Tripoli Medical University, New Orleans, USA; 2 General Medicine, Alexandria Medical Associates, Alexandria, USA; 3 Internal Medicine, Bingham University College of Health Sciences, Jos, NGA; 4 Hospital Medicine, Mercy Medical Center, Cedar Rapids, USA; 5 Internal Medicine, Independent Physician and Researcher, Westland, USA; 6 Internal Medicine, Interfaith Medical Center, New York, USA; 7 Family Medicine, Abia State University, Aba, NGA; 8 Mental Health, Braxia Health, Mississauga, CAN; 9 Medicine, University of Nigeria College of Medicine, Enugu, NGA; 10 Public Health, Mercer University Health Sciences Center, Atlanta, USA; 11 Medicine and Surgery, University of Tripoli, Tripoli, LBY; 12 Gastroenterology and Hepatology, Lagos University Teaching Hospital, Lagos, NGA

**Keywords:** blood pressure control, cardiovascular disease, diabetes mellitus, glp-1 receptor agonists, hypertension, nhanes

## Abstract

Background: Hypertension remains a major contributor to cardiovascular morbidity and mortality in the United States. Glucagon-like peptide 1 receptor agonists (GLP-1 RAs) are increasingly used in the management of type 2 diabetes and obesity, but population-level data describing blood pressure control among GLP-1 RA users with hypertension remain limited.

Objective: To describe differences in blood pressure control between GLP-1 RA users and non-users among U.S. adults with hypertension using data from the National Health and Nutrition Examination Survey (NHANES) 2017 to March 2020.

Methods: This cross-sectional study analyzed NHANES 2017 to March 2020 pre-pandemic data. Adults aged 18 years and older with hypertension were included. Current GLP-1 RA use was identified from prescription medication records. Blood pressure control was defined as mean systolic blood pressure <130 mmHg and mean diastolic blood pressure <80 mmHg. Survey weights, strata, and primary sampling units were applied to account for the complex sampling design. Descriptive statistics, survey-adjusted t tests, and survey-adjusted chi-square tests were used for group comparisons.

Results: The final analytic sample included 4,016 adults with hypertension, of whom only 45 participants reported GLP-1 RA use. After applying NHANES sampling weights, GLP-1 RA users represented an estimated 994,028 (0.9%) U.S. adults, while non-users represented 104,940,242 (99.1%). The prevalence of controlled blood pressure was higher among GLP-1 RA users than non-users (659,630 (66%) vs. 24,950,382 (24%); design-adjusted *F *= 15.22, *P *= 0.001). GLP-1 RA users had a higher mean body mass index than non-users (37.25 ± 7.41 vs. 31.22 ± 7.22 kg/m², *P *= 0.008). Diabetes (988,214 (99%) vs. 18,545,714 (18%), *P *< 0.001)) and cardiovascular disease history (233,659 (24%) vs. 16,122,408 (15%), *P *= 0.035) were also more common among GLP-1 RA users.

Conclusions: Among U.S. adults with hypertension, GLP-1 RA users demonstrated a higher prevalence of blood pressure control than non-users despite a greater burden of obesity, diabetes, and cardiovascular disease. These findings highlight the importance of continued investigation of blood pressure patterns among individuals receiving GLP-1 RAs in contemporary clinical practice.

## Introduction

Hypertension is a leading chronic disease that affects nearly 50% of adults in the United States and has become a leading contributor to the morbidity, mortality, and medical costs for cardiovascular disease (CVD) [1,2]. Despite continued improvements in screening, diagnosis, and treatment of hypertension, only a relatively small percentage of U.S. adults with hypertension are successfully controlling their blood pressure [3]. Individuals who do not adequately control their blood pressure are at greater risk of developing coronary artery disease (CAD), stroke, heart failure, chronic kidney disease (CKD), peripheral vascular disease (PVD), and premature death [4,5]. As obesity, type 2 diabetes, and metabolic syndrome continue to increase within the U.S. population, the total burden of hypertension and its complications will likely remain a significant public health issue [6].

Historically, the management of hypertension has primarily consisted of lifestyle change and antihypertensive pharmacotherapy as methods to achieve blood pressure control. More recently, however, the focus has begun to shift toward therapies that have both cardiometabolic and cardiovascular benefits beyond just controlling blood glucose or reducing weight [7]. One class of such therapies includes glucagon-like peptide 1 receptor agonists (GLP-1 RAs), which were developed originally for use in patients with type 2 diabetes and have now gained recognition as potential agents for obesity management and cardiovascular risk reduction [8,9].

GLP-1 RAs (semaglutide, liraglutide, and dulaglutide) are effective in improving glycemic control and achieving clinically significant weight loss in adults with obesity and diabetes, and they have also shown beneficial effects on a number of cardiovascular risk factors (particularly blood pressure) in clinical trials [10,11]. While several mechanisms have been proposed for how GLP-1 RAs may affect cardiovascular risk factors, such as increasing natriuresis, reducing sympathetic nervous system activity, improving endothelial function, reducing vascular inflammation, and improving arterial stiffness, likely all of these mechanisms work together to contribute to the improved regulation of blood pressure and overall cardiovascular health [12]. In recent years, large-scale, randomized controlled trials (RCTs) have demonstrated that GLP-1 RAs reduce the incidence of major cardiovascular events in carefully selected patients at high risk for these events [13,14]. As interest in GLP-1 RAs has increased, it has become apparent that there may be significant potential for GLP-1 RAs to help improve hypertension in routine clinical practice [15,16]. Because hypertension is often present in patients with diabetes and obesity, clinical interventions that can improve multiple cardiometabolic risk factors at once are of particular importance in terms of their impact on the health of the population and the prevention of CVDs [17].

Although studies conducted using RCT designs have shown that treatment with GLP-1 RAs can significantly lower blood pressure, no representative results exist for the population of the United States that clearly demonstrate how well GLP-1 RAs work to lower blood pressure among adults aged 18 or older who have hypertension [18]. Participants in clinical trials may not always be representative of the general population due to a variety of factors, including differences in demographics, socioeconomic status, access to healthcare, comorbidity burden, and compliance with prescribed medications [19], thus limiting the generalizability of the results from any clinical trial. To better evaluate the real-world effect of GLP-1 RAs on blood pressure control among people living with hypertension in the United States, it is necessary for researchers to conduct more robust, population-based studies.

The National Health and Nutrition Examination Surveys (NHANES) offer an opportunity for researchers to use data collected from the civilian noninstitutionalized population of the United States to investigate these effects using data collected by the NHANES from 2017 to 2020 [20]. The NHANES database contains detailed demographic, clinical, laboratory, examination, and prescription medication data, allowing for a thorough evaluation of CVD risk factors and medication usage patterns in the United States. Therefore, this study aims to describe differences in the prevalence of blood pressure control between GLP-1 RA users and non-users among U.S. adults with hypertension using NHANES 2017-March 2020 data.

## Materials and methods

Study design and data source

This study employed a cross-sectional analytical design using data from NHANES 2017 to March 2020, the pre-pandemic cycle [21]. NHANES is a nationally representative survey conducted by the National Center for Health Statistics of the Centers for Disease Control and Prevention. The survey uses a complex, multistage probability sampling design to collect information on the health and nutritional status of the civilian, noninstitutionalized U.S. population through household interviews, physical examinations, and laboratory assessments. The 2017 to March 2020 pre-pandemic data release combines information collected before suspension of field operations in March 2020 and provides nationally representative estimates for the U.S. population during this period.

Study population

**Figure 1:**
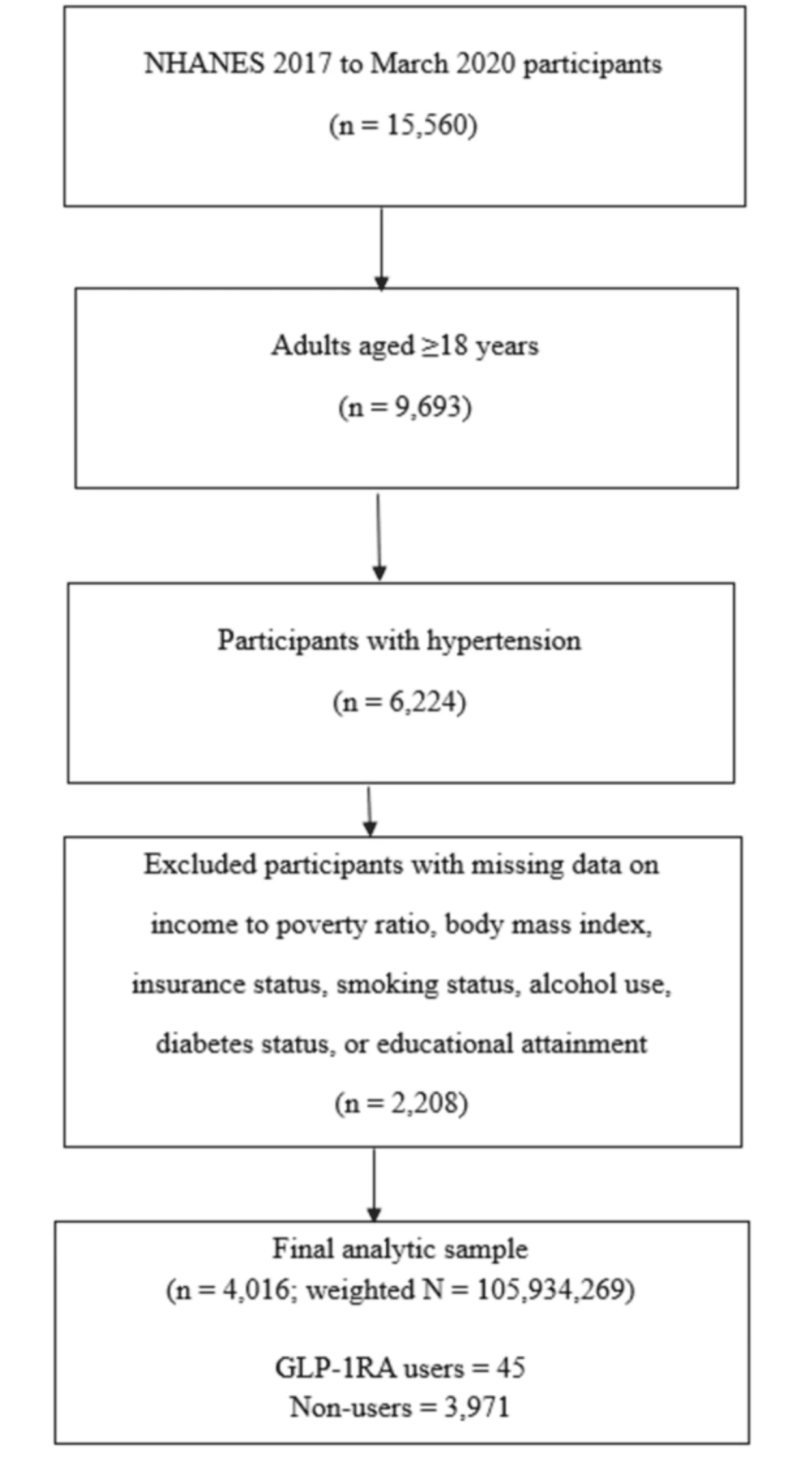
Flow diagram of participant selection, NHANES 2017 to March 2020. Image created by authors. GLP-1 RA, glucagon-like peptide 1 receptor agonist; NHANES, National Health and Nutrition Examination Survey

The study population comprised adults aged 18 years or older with hypertension. Participants were initially identified from the NHANES demographic file and subsequently linked across questionnaire, examination, laboratory, and prescription medication datasets using the unique respondent identifier (SEQN). Hypertension was defined as a self-reported history of hypertension or current use of antihypertensive medication. Participants with missing information on variables included in the final analysis were excluded through complete case analysis. The final analytic sample included 4,016 participants, representing an estimated weighted population of 105,934,269 U.S. adults with hypertension. Among the final analytic sample, 45 participants reported current use of a GLP-1 RA, as shown in Figure 1.

Variables and measures

The primary exposure variable was current GLP-1 RA use. Prescription medication records were reviewed to identify participants reporting use of semaglutide, liraglutide, dulaglutide, exenatide, or lixisenatide during the previous month. Participants reporting any of these medications were classified as GLP-1 RA users.

The primary outcome was blood pressure control. Blood pressure measurements were obtained during the NHANES mobile examination center visit using standardized protocols. Up to three systolic and diastolic blood pressure measurements were available for each participant. Mean systolic and mean diastolic blood pressure values were calculated using available measurements. Blood pressure control was defined as a mean systolic blood pressure of less than 130 mmHg and a mean diastolic blood pressure of less than 80 mmHg. Participants meeting both criteria were classified as having controlled blood pressure.

Covariates were selected based on clinical relevance and availability within NHANES. Demographic variables included age, sex, race and ethnicity, educational attainment, and family income-to-poverty ratio. Race and ethnicity were categorized as Mexican American, Other Hispanic, Non-Hispanic White, Non-Hispanic Black, Non-Hispanic Asian, or Other Race. Educational attainment was categorized as less than high school, high school graduate or general education development (GED), and college or above. Clinical characteristics included body mass index, diabetes status, CVD history, and health insurance coverage. Diabetes status was based on self-reported physician diagnosis of diabetes. CVD history was defined by a self-reported history of congestive heart failure, coronary heart disease, angina, myocardial infarction, or stroke. Lifestyle factors included smoking status, alcohol use, and physical activity. Smoking status was classified using cigarette smoking history and current smoking behavior. Alcohol use was categorized as current alcohol use or no current alcohol use. Physical activity was categorized as active or inactive based on reported participation in moderate or vigorous occupational or recreational activities.

Missing data

Missing data were evaluated for all variables considered for analysis. Missing data were observed for the family income-to-poverty ratio (16.97%), body mass index (14.15%), insulin use (80.30%), health insurance coverage (0.24%), smoking status (0.06%), alcohol use (19.33%), diabetes status (3.39%), and educational attainment (2.17%). Mean systolic and mean diastolic blood pressure each had 26.82% missingness; however, these variables were used only to derive the blood pressure control outcome and were therefore not included in the complete-case restriction. Insulin use demonstrated substantial structural missingness because the variable was collected only among participants with diabetes. Inclusion of insulin use would have resulted in a substantial reduction in sample size and potential selection bias; therefore, it was excluded from the final analyses. Complete-case analysis was subsequently applied to participants with available information for all variables included in the final analytic models and descriptive comparisons. This approach resulted in the exclusion of participants with incomplete data, which may have introduced selection bias if those excluded differed systematically from those included in the analysis.

Statistical analysis

All analyses accounted for the complex NHANES sampling design using examination sample weights, masked variance strata, and primary sampling units. Survey weights specific to the 2017 to March 2020 pre-pandemic cycle were applied to generate nationally representative estimates. Continuous variables were summarized as means and standard deviations, while categorical variables were summarized as weighted frequencies and column percentages.

Descriptive comparisons were conducted according to GLP-1 RA use status. Differences in continuous variables were evaluated using survey-adjusted t-tests, while differences in categorical variables were assessed using survey-adjusted chi-square tests. The primary analysis focused on describing differences in blood pressure control between GLP-1 RA users and non-users. Multivariable regression analysis was considered during study planning; however, only 45 participants in the final analytic sample reported GLP-1 RA use. Given the limited number of exposed participants, multivariable modeling was not performed because estimates were expected to be unstable and potentially unreliable. The analysis therefore focused on survey-weighted descriptive and bivariate comparisons. All analyses were performed using Stata version 18 (StataCorp, College Station, TX) [22].

Ethical considerations

NHANES protocols were reviewed and approved by the National Center for Health Statistics Research Ethics Review Board, and written informed consent was obtained from all participants before data collection. The present study used publicly available, deidentified data and did not involve direct contact with participants. As a secondary analysis of anonymized publicly accessible data, additional institutional review board approval was not required.

## Results

Table 1 presents the demographic, socioeconomic, clinical, and behavioral characteristics of U.S. adults with hypertension according to GLP-1 RA use.

**Table 1:** Characteristics of U.S. adults with hypertension according to GLP-1 receptor agonist use, NHANES 2017-March 2020. Values are presented as weighted population estimates and weighted column percentages for categorical variables, and weighted mean (SD) for continuous variables. Continuous variables were compared using survey-adjusted t-tests. Categorical variables were compared using survey-adjusted chi-square tests. All estimates account for NHANES sampling weights, strata, and primary sampling units. The asterisk (*) indicates statistical significance at *P *< 0.05. The symbol (-) represents intentionally left blank. The table was generated by the authors using Stata version 18 [22]. GLP-1 RA, glucagon-like peptide 1 receptor agonist; CVD, cardiovascular disease; GED, general educational development; NHANES, National Health and Nutrition Examination Survey

Characteristic	No GLP-1 RA use (*N* = 104,940,242)	GLP-1 RA use (*N* = 994,028)	Test statistic	*P*-value
Age (years), mean ± SD	54.25 ± 16.26	56.82 ± 11.98	*t* = -1.20	0.241
Body mass index (kg/m²), mean ± SD	31.22 ± 7.22	37.25 ± 7.41	*t* = -2.87	0.008*
Income-to-poverty ratio, mean ± SD	3.12 ± 1.60	3.01 ± 1.66	*t* = 0.40	0.695
Sex, *n* (%)	-	-	*F* = 2.01	0.168
Male	53,645,822 (51%)	636,809 (64%)	-	-
Female	51,294,420 (49%)	357,219 (36%)	-	-
Race/Ethnicity, *n* (%)	-	-	*F* = 0.99	0.388
Mexican American	6,359,239 (6%)	78,317 (8%)	-	-
Other Hispanic	6,730,254 (6%)	37,636 (4%)	-	-
Non-Hispanic White	69,486,345 (66%)	723,355 (73%)	-	-
Non-Hispanic Black	12,983,584 (12%)	128,927 (13%)	-	-
Non-Hispanic Asian	4,803,755 (6%)	4,026 (0.4%)	-	-
Other race	4,577,065 (4%)	21,767 (1.6%)	-	-
Education level, *n* (%)	-	-	*F* = 0.61	0.507
Less than high school	11,562,025 (11%)	82,704 (8%)	-	-
High school graduate/GED	30,792,914 (29%)	387,553 (39%)	-	-
College+	62,585,303 (60%)	523,771 (53%)		
Diabetes status, *n* (%)	-	-	*F* = 708.16	<0.001*
No diabetes	86,394,528 (82%)	5,814 (1%)	-	-
Diabetes	18,545,714 (18%)	988,214 (99%)	-	-
Smoking status, *n* (%)	-	-	*F* = 1.24	0.297
Never smoker	54,243,802 (52%)	497,304 (50%)	-	-
Former smoker	33,347,179 (32%)	240,013 (24%)	-	-
Current smoker	17,349,261 (16%)	256,711 (26%)	-	-
Alcohol consumption, *n* (%)	-	-	*F* = 0.26	0.614
Non-drinker	7,117,065 (7%)	47,011 (5%)	-	-
Drinker	97,823,177 (93%)	947,017 (95%)	-	-
Physical activity, *n* (%)	-	-	*F* = 0.86	0.362
Inactive	28,966,010 (28%)	230,805 (23%)	-	-
Active	75,974,232 (72%)	763,223 (77%)	-	-
History of cardiovascular disease, *n* (%)	-	-	*F* = 4.97	0.035*
No CVD history	88,817,834 (85%)	760,369 (76%)	-	-
CVD history	16,122,408 (15%)	233,659 (24%)	-	-
Health insurance coverage, *n* (%)			*F* = 1.96	0.174
Uninsured	11,046,590 (11%)	0 (0%)		
Insured	93,893,652 (89%)	994,028 (100%)		
Blood pressure control (<130/80 mmHg), *n* (%)			*F* = 15.22	0.001*
Uncontrolled	79,989,860 (76%)	334,398 (34%)		
Controlled	24,950,382 (24%)	659,630 (66%)		

The final analytic sample represented an estimated 105,934,269 U.S. adults with hypertension, including 104,940,242 (99.1%) non-users of GLP-1 RAs and 994,028 (0.9%) GLP-1 RA users. The mean age was similar between non-users and users, 54.25 (16.26) years and 56.82 (11.98) years, respectively (*P *= 0.241). Body mass index was higher among GLP-1 RA users than non-users, 37.25 (7.41) kg/m² versus 31.22 (7.22) kg/m² (*P* = 0.008). No difference was observed for income-to-poverty ratio between the two groups (*P* = 0.695).

Among non-users, 53,645,822 (51%) were male and 51,294,420 (49%) were female. Among GLP-1 RA users, 636,809 (64%) were male and 357,219 (36%) were female. The distribution of sex did not differ between groups (*P* = 0.168). Race and ethnicity distributions were also comparable between groups (*P* = 0.388). Non-Hispanic White participants represented the largest racial and ethnic group among both non-users (69,486,345, 66%) and GLP-1 RA users (723,355, 73%).

Educational attainment was similar between groups (*P* = 0.507). Among non-users, 11,562,025 (11%) had less than a high school education, 30,792,914 (29%) were high school graduates or had a GED, and 62,585,303 (60%) had completed college or higher education. Among GLP-1 RA users, 82,704 (8%) had less than a high school education, 387,553 (39%) were high school graduates or had a GED, and 523,771 (53%) had completed college or higher education.

A marked difference was observed for diabetes status (*P *< 0.001). Among GLP-1 RA users, 988,214 (99%) had diabetes, compared with 18,545,714 (18%) among non-users. History of CVD was also more common among GLP-1 RA users (233,659, 24%) than among non-users (16,122,408, 15%) (*P* = 0.035).

Smoking status, alcohol consumption, physical activity, and health insurance coverage did not differ significantly between groups. Current smokers accounted for 256,711 (26%) of GLP-1 RA users and 17,349,261 (16%) of non-users (*P* = 0.297). Current alcohol use was reported by 947,017 (95%) GLP-1 RA users and 97,823,177 (93%) non-users (*P* = 0.614). Active physical activity was reported by 763,223 (77%) GLP-1 RA users and 75,974,232 (72%) non-users (*P* = 0.362). Nearly all GLP-1 RA users were insured, representing 994,028 (100%) of users, compared with 93,893,652 (89%) among non-users (*P* = 0.174).

Blood pressure control differed significantly according to GLP-1 RA use (*P* = 0.001). Controlled blood pressure was observed among 659,630 (66%) GLP-1 RA users compared with 24,950,382 (24%) non-users. Conversely, uncontrolled blood pressure was present among 334,398 (34%) GLP-1 RA users and 79,989,860 (76%) non-users.

## Discussion

This study described differences in blood pressure control between GLP-1 RA users and non-users among U.S. adults with hypertension using NHANES 2017 to March 2020 data. A higher prevalence of blood pressure control was observed among GLP-1 RA users than among non-users. GLP-1 RA users also had higher body mass index values and were more likely to have diabetes and a history of CVD. No significant differences were observed for age, sex, race and ethnicity, educational attainment, smoking status, alcohol use, physical activity, income-to-poverty ratio, or health insurance coverage. These findings are noteworthy because blood pressure control remains a major public health challenge in the United States despite advances in prevention and treatment strategies. National estimates have shown that a substantial proportion of adults with hypertension do not achieve recommended blood pressure targets [1,3]. The higher prevalence of blood pressure control observed among GLP-1 RA users should be interpreted cautiously because this cross-sectional study cannot establish temporal or causal relationships. The observed differences may reflect variations in patient characteristics, prescribing patterns, or clinical management rather than an effect of GLP-1 RA use itself. In particular, confounding by indication is likely, as nearly all GLP-1 RA users had diabetes and a greater proportion had CVD, conditions for which these medications were commonly prescribed during the study period. These individuals may also have received more intensive cardiovascular risk management and closer clinical follow-up, which could have contributed to the higher prevalence of blood pressure control observed in this study. The finding that nearly all GLP-1 RA users had diabetes is consistent with the therapeutic indications of these agents during the study period, when their use was largely concentrated among individuals with type 2 diabetes [7].

Current U.S. guidelines emphasize achieving recommended blood pressure targets through comprehensive cardiovascular risk management, particularly among individuals with diabetes and obesity [4,7,16,22,23]. The higher prevalence of controlled blood pressure observed among GLP-1 RA users is consistent with the fact that these medications were primarily prescribed to patients with diabetes and elevated cardiovascular risk during the study period. However, because of the cross-sectional design, these findings should be interpreted as descriptive and not as evidence that GLP-1 RAs improve blood pressure control.

Several biological and clinical pathways may help explain the observed differences in blood pressure control. GLP-1 RAs are known to influence multiple physiological processes beyond glucose regulation, including appetite regulation, body weight, gastric emptying, and cardiometabolic function [8]. Clinical trials have consistently demonstrated meaningful reductions in body weight among individuals receiving GLP-1 RAs, particularly semaglutide [9,10]. In the present study, GLP-1 RA users had substantially higher body mass index values than non-users, suggesting that these medications were being used among individuals with a greater burden of obesity. Weight reduction may contribute to improvements in blood pressure through reductions in sympathetic activity, vascular resistance, and metabolic stress [14]. Experimental and clinical evidence has also suggested that GLP-1 RAs may be associated with modest reductions in blood pressure independent of weight loss [8,13]. In a randomized crossover study among patients with type 2 diabetes and CAD, liraglutide treatment was associated with reductions in ambulatory systolic blood pressure measurements [13]. Although the present study was not designed to evaluate treatment effects, these observations provide clinical context for the differences identified in the descriptive analyses.

The higher prevalence of diabetes and CVD among GLP-1 RA users is also consistent with contemporary prescribing patterns and evidence from cardiovascular outcome trials. Large randomized trials involving liraglutide, semaglutide, and dulaglutide have demonstrated cardiovascular benefits among individuals with type 2 diabetes who are at increased cardiovascular risk [11,12,17]. As a result, these medications are frequently prescribed to patients with diabetes who have established CVD or multiple cardiovascular risk factors [7,16]. The greater proportion of participants with CVD among GLP-1 RA users in this study likely reflects these clinical indications. Despite having a higher burden of obesity, diabetes, and CVD, GLP-1 RA users exhibited a greater prevalence of blood pressure control. This pattern highlights the importance of considering the broader cardiometabolic profile of patients receiving these therapies.

Strengths and limitations of the study

This study used nationally representative NHANES data and accounted for the complex survey design, allowing the findings to reflect U.S. adults with hypertension during the study period [18-20]. Several limitations should be considered. GLP-1 RA use was uncommon between 2017 and March 2020, leaving only 45 exposed participants in the final analytic sample. Because of the small exposed group and the number of clinically relevant covariates available, multivariable regression was not performed due to concerns regarding unstable estimates and overfitting. The findings should therefore be interpreted as descriptive comparisons rather than adjusted associations. Furthermore, NHANES 2017 to March 2020 represents a period when GLP-1 RAs were used predominantly for type 2 diabetes, before their widespread adoption for obesity management and broader cardiovascular risk reduction. Consequently, the findings may not fully reflect current prescribing patterns or contemporary clinical practice.

Complete-case analysis reduced the sample from 6,224 to 4,016 participants because of missing information for variables such as alcohol use, income-to-poverty ratio, and body mass index. Although this approach ensured complete information for the analyses, it may have introduced selection bias if participants with missing data differed systematically from those included in the final analytic sample. Insulin use was not included in the final analysis because more than 80% of observations were missing, largely because the variable applied only to participants with diabetes. Information on antihypertensive treatment, medication adherence, duration of hypertension, duration of GLP-1 RA use, dietary factors, and healthcare utilization was not available in the final analytic dataset and could not be evaluated. In addition, nearly all GLP-1 RA users had diabetes and a higher prevalence of CVD, raising the possibility of confounding by indication and differences in clinical management that could not be accounted for in the descriptive analyses. Several variables also relied on self-report and may be subject to reporting error. Finally, the cross-sectional design precludes assessment of temporal relationships or causal inference between GLP-1 RA use and blood pressure control. Future studies using more recent data and larger numbers of GLP-1 RA users may help clarify these patterns through appropriately adjusted analyses.

## Conclusions

This study described differences in blood pressure control between GLP-1 RA users and non-users among U.S. adults with hypertension using nationally representative NHANES data. Participants using GLP-1 RAs showed a higher prevalence of blood pressure control despite having a greater burden of obesity, diabetes, and CVD. These findings add to the growing interest in the broader cardiometabolic profile of individuals receiving GLP-1 RAs in routine clinical practice. Blood pressure control remains a key component of cardiovascular risk reduction, particularly among adults with multiple chronic conditions. Further research using larger and more recent populations of GLP-1 RA users is needed to better characterize blood pressure patterns and evaluate these findings in settings where use of these therapies has become more common.

## References

[1] Muntner P, Hardy ST, Fine LJ, Jaeger BC, Wozniak G, Levitan EB, Colantonio LD (2020). Trends in blood pressure control among US adults with hypertension, 1999-2000 to 2017-2018. JAMA.

[2] Tsao CW, Aday AW, Almarzooq ZI (2023). Heart disease and stroke statistics-2023 update: A report from the American Heart Association. Circulation.

[3] Fryar CD, Ostchega Y, Hales CM, Zhang G, Kruszon-Moran D (2017). Hypertension prevalence and control among adults: United States, 2015-2016. NCHS Data Brief.

[4] Whelton PK, Carey RM, Aronow WS (2018). 2017 ACC/AHA/AAPA/ABC/ACPM/AGS/APhA/ASH/ASPC/NMA/PCNA guideline for the prevention, detection, evaluation, and management of high blood pressure in adults: a report of the American College of Cardiology/American Heart Association Task Force on Clinical Practice Guidelines. Hypertension.

[5] Virani SS, Alonso A, Aparicio HJ (2021). Heart disease and stroke statistics-2021 update: A report from the American Heart Association. Circulation.

[6] Hales CM, Carroll MD, Fryar CD, Ogden CL (2020). Prevalence of obesity and severe obesity among adults: United States, 2017-2018. NCHS Data Brief.

[7] (2024). Pharmacologic approaches to glycemic treatment: standards of care in diabetes-2024. Diabetes Care.

[8] Drucker DJ (2018). Mechanisms of action and therapeutic application of glucagon-like peptide-1. Cell Metab.

[9] Rubino D, Abrahamsson N, Davies M (2021). Effect of continued weekly subcutaneous semaglutide vs placebo on weight loss maintenance in adults with overweight or obesity: the STEP 4 randomized clinical trial. JAMA.

[10] Wilding JP, Batterham RL, Calanna S (2021). Once-weekly semaglutide in adults with overweight or obesity. N Engl J Med.

[11] Marso SP, Daniels GH, Brown-Frandsen K (2016). Liraglutide and cardiovascular outcomes in type 2 diabetes. N Engl J Med.

[12] Marso SP, Bain SC, Consoli A (2016). Semaglutide and cardiovascular outcomes in patients with type 2 diabetes. N Engl J Med.

[13] Kumarathurai P, Anholm C, Fabricius-Bjerre A (2017). Effects of the glucagon-like peptide-1 receptor agonist liraglutide on 24-h ambulatory blood pressure in patients with type 2 diabetes and stable coronary artery disease: a randomized, double-blind, placebo-controlled, crossover study. J Hypertens.

[14] Perdomo CM, Cohen RV, Sumithran P (2023). Contemporary medical, device, and surgical therapies for obesity in adults. Lancet.

[15] Sattar N, McGuire DK (2018). Pathways to cardiorenal complications in type 2 diabetes mellitus: a need to rethink. Circulation.

[16] (2024). Cardiovascular disease and risk management: Standards of care in diabetes-2024. Diabetes Care.

[17] Gerstein HC, Colhoun HM, Dagenais GR (2019). Dulaglutide and cardiovascular outcomes in type 2 diabetes (REWIND): a double-blind, randomised placebo-controlled trial. Lancet.

[18] Akinbami LJ, Chen TC, Davy O (2022). National Health and Nutrition Examination Survey, 2017-March 2020 prepandemic file: sample design, estimation, and analytic guidelines. Vital Health Stat 1.

[19] Johnson CL, Dohrmann SM, Burt VL, Mohadjer LK (2014). National health and nutrition examination survey: sample design, 2011-2014. Vital Health Stat 2.

[20] Mirel LB, Mohadjer LK, Dohrmann SM, Clark J, Burt VL, Johnson CL, Curtin LR (2013). National Health and Nutrition Examination Survey: estimation procedures, 2007-2010. Vital Health Stat 2.

[21] (2026). National Health and Nutrition Examination Survey (NHANES). https://wwwn.cdc.gov/nchs/nhanes/search/default.aspx.

[22] StataCorp. StataCorp. (2026). Stata Statistical Software: Release 18. https://www.stata.com/stata18/.

[23] Goupil R, Tsuyuki RT, Santesso N (2025). Hypertension Canada guideline for the diagnosis and treatment of hypertension in adults in primary care. CMAJ.

